# A Case of Old Calcifying Epithelioma Processed Symptomless over 40 Years

**DOI:** 10.1155/2013/572372

**Published:** 2013-08-04

**Authors:** Shinji Yamaguchi, Madoka Inui, Takashi Takeoka, Kenya Okumura, Toshiro Tagawa

**Affiliations:** Department of Oral and Maxillofacial Surgery, Department of Clinical Sciences, Medical Life Sciences, Mie University, Graduate School of Medicine, 2-174 Edobashi, Tsu, Mie 514-8507, Japan

## Abstract

Calcifying epithelioma, a benign tumor derived from the hair apparatus and consisted of hair matrix cells, is relatively prevalent in females. We report a case of right preauricular calcifying epithelioma that was incidentally detected at the examination of multiple facial fractures and became an old lesion without symptoms for 40 years. The patient who was a 42-year-old male visited our department for the first time in October 2011 with a chief complaint of multiple facial fractures. Radiographic imaging demonstrated fracture lines at the anterior and posterior walls of the left maxillary sinus and zygomatic arch and revealed a mass at a right preauricular area. The extraction was performed under general anaesthesia. No recurrence has been observed 15 months after surgery. We also reviewed the literature of calcifying epithelioma.

## 1. Introduction

Calcifying epithelioma, a benign tumor derived from the hair apparatus and consisted of hair matrix cells, is relatively prevalent in females. It often develops on the face, neck, and upper limbs in the youth and is palpable as a hard nodule under the skin. Pathologically, it is mostly consisted of eosinophils and shadow cells, and the proportion of shadow cells increases over time, which leads to calcification and osteogenesis [1].

 Although there are a number of reports on this tumor from the dermatology, reporting from the oral and maxillofacial region is relatively rare. We here report a case of calcifying epithelioma that was incidentally detected at the examination of multiple facial fractures and became an old lesion without symptoms for 40 years. 

## 2. Case

A 42-year-old male visited our department for the first time in October 2011 with a chief complaint of multiple facial fractures. He had no past history or family history. As for the present history, he fell down from a bicycle and was hit severely in his left face in October 2011. He visited a nearby general hospital and was diagnosed with facial fractures. Then, he was referred to our department. 

**Figure 1:**
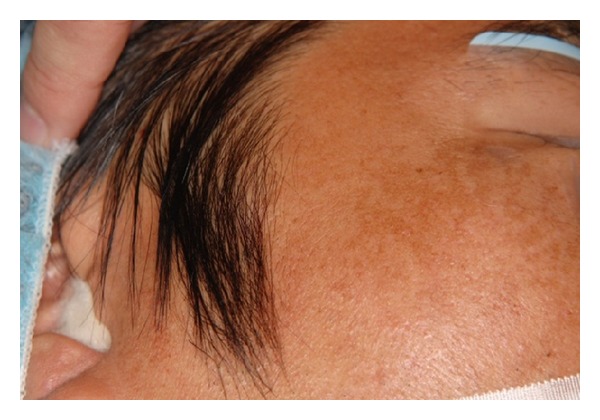
Revealed mass at the right preauricular area.

Radiographic imaging demonstrated fracture lines at the anterior and posterior walls of the left maxillary sinus and zygomatic arch and revealed a mass at a right preauricular area, well delineated radiopaque (Figure 1).

 History taking revealed that his mother had noticed the mass when he was three years old, but there was no symptom. Since he hoped extraction, the right preauricular mass was resected at the same time with reduction and fixation of multiple facial fractures under general anesthesia. 

**Figure 2:**
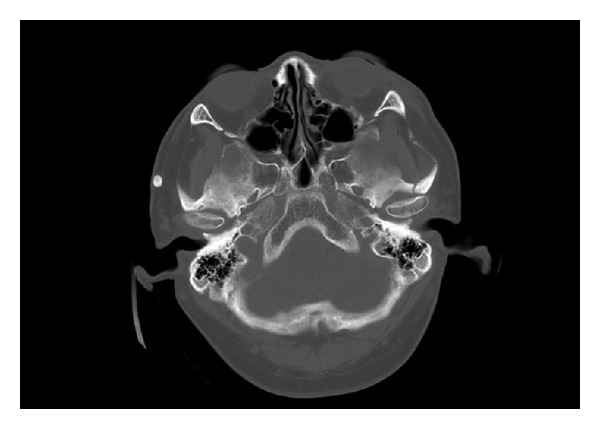
A bone-like hard mass about 10 mm in size was palpable subcutaneously at the right preauricular area.

 General physical examination revealed average body constitution with excellent nutritious status and no abnormality. Extraoral findings revealed a depression at the cheek on the left, anesthesia, and palpebral subconjunctival hemorrhage on the left, but there was no motility disorder of the left eyeball, double vision, or occlusal deviation. A bone-like hard mass about 10 mm in size was palpable subcutaneously at the right preauricular area. The mass was well delineated without adhesion to the adjacent tissue. The surface of the skin showed a healthy color, and the mass developed no symptom or growing tendency from the childhood (Figure 2). 

 In the oral cavity, there was no abnormal finding, and the excretion and nature of saliva were normal. 

Tentative diagnosis of maxillary fracture and preauricular benign tumor was made, and the right preauricular mass was resected at the same time with open reduction and fixation of multiple left facial fractures. A horizontal skin incision was made about 1 cm from the top of the mass, and the mass was dissected from the adjacent tissue. The mass had no adhesion with the adjacent tissue, and it was resected *en bloc*. 

**Figure 3:**
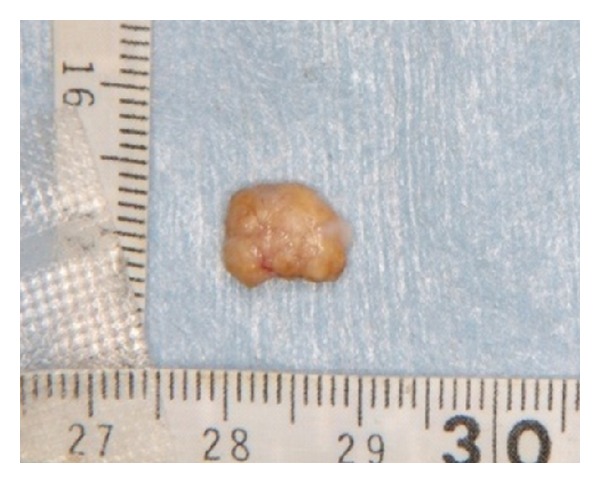
The excised specimen was hard and yellowish white 10 × 9 mm in size, and the cross section showed a coarse and bone-like surface.

**Figure 4:**
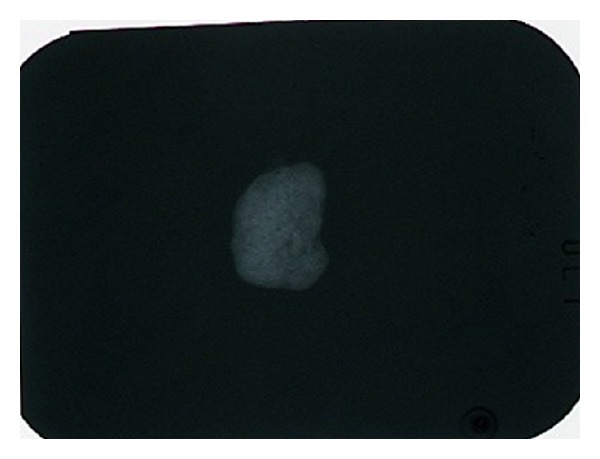
Dental X-rays showed an image of patchy calcification in the mass.

 The resected specimen was hard and yellowish white 10 × 9 mm in size, and the cross section showed a coarse and bone-like surface (Figure 3). Dental X-rays showed an image of patchy calcification in the mass (Figure 4). 

## 3. Pathological Findings

**Figure 5:**
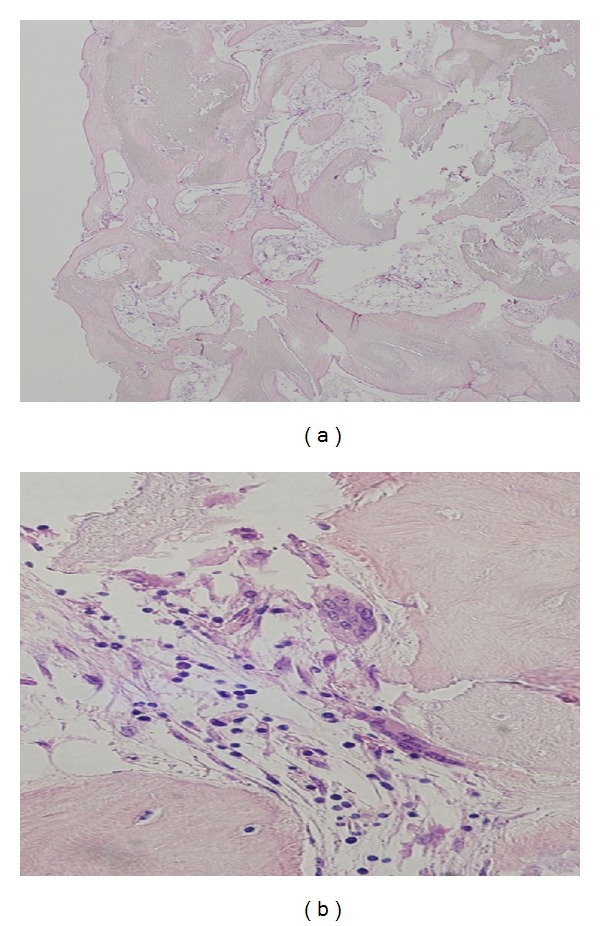
Pathological findings 1 (H-E, ×40 and 400): aggregation of shadow cells and surrounded bone tissue were observed. At the stroma, multinuclear giant cells are sparsely observed, and there was no infiltration of eosinophils. Old calcifying epithelioma with ossification was diagnosed.

Aggregation of shadow cells and surrounded bone tissue were observed. At the stroma, multinuclear giant cells are sparsely observed, and there was no infiltration of eosinophils. Old calcifying epithelioma with ossification was diagnosed (Figures 5(a) and 5(b)).

## 4. Discussion

Calcifying epithelioma, mostly consisted of eosinophils, shadow cells, and transitional cells, exhibits a variety of images of calcareous deposits, bone formation, foreign-body giant cells, and melanin deposits. It is known that the component ratio of eosinophils to shadow cells in this tumor changes as disease stages advance and tumor parenchyma is rich in eosinophils at the early stage, whereas shadow cells are predominant in the old tumor with almost no eosinophil infiltration [2, 3]. Moreover, the tumor is characterized by calcification, but calcification is not always accompanied. Calcification is mostly observed in shadow cells, and it is considered dystrophic calcification associated with degeneration and necrosis of shadow cells [1]. 

 We experienced a case of calcifying epithelioma that was incidentally detected as a circle-like radiopaque finding by CT in a patient with multiple facial fractures and diagnosed by history taking as an old lesion without symptoms for about 40 years. The prognosis of the tumor is generally excellent, but recurrent cases have been reported [1, 4], and malignant change after repeated recurrences has also been reported [5, 6]. In our case, despite a short postoperative follow-up period of about fifteen months, no recurrence has been observed. 

 Moehlenbeck [7] mentioned that this tumor occurred at younger than 10 years old in more than 40% of 900 cases examined at the dermatology department, and patients developed symptoms in their 30s or at younger age in more than 60% with an average period of 4.4 years from appearance to extraction. Cases without symptoms for a long time, such as this case for 40 years, are rare. 

**Figure 6:**
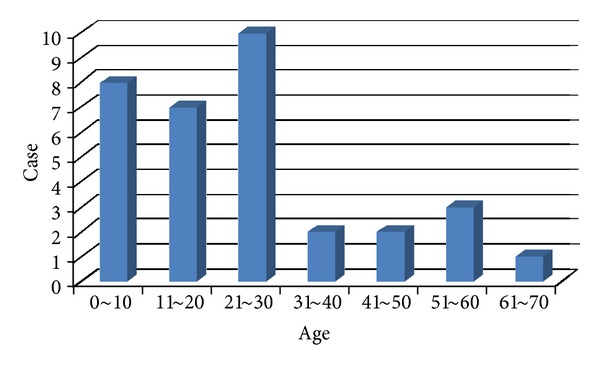


**Figure 7:**
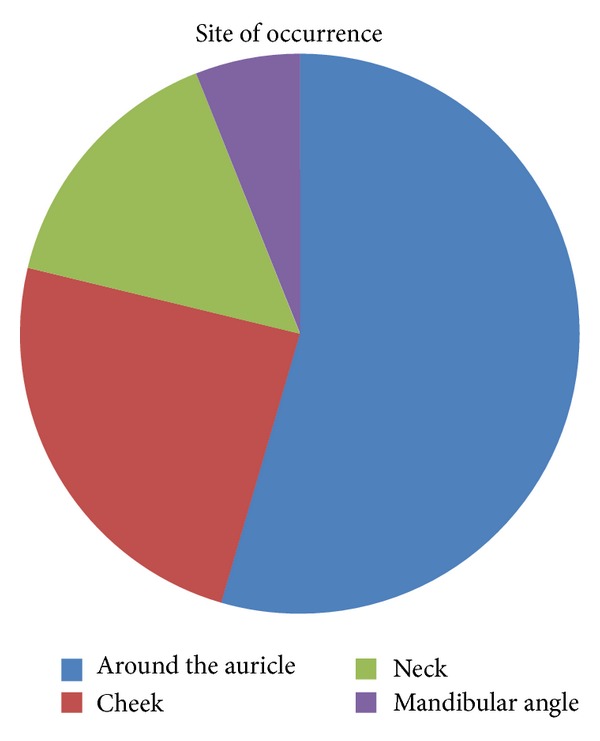


 In Japan, 33 cases of calcifying epithelioma in the oral and maxillofacial region were reported between 1978 and 2012 including our case [2–4, 8–24]. The age distributed from 4 to 68 years old, and the average age was 23.8 years. The most frequent site of occurrence is periauricular in 18 cases, followed by the cheek in 8, neck in 5, and angle of the mandible in 2. Disease duration ranged from 1 month to 40 years with the average of 5.8 years. The tumor diameter ranged from 4 mm to 50 mm with the average of 22.1 mm (Table 1, Figures 6 and 7).

 Pathologically, there were 9 cases with only shadow cells without eosinophils, 13 with bone formation, and 17 with giant cells, while there were only 3 cases with only shadow cells accompanied by bone formation and giant cells, including our case. 

 Then, we focused on the occurrence of the tumor and the period of its presence *in vivo*. However, since it is impossible to clearly determine the period of its presence *in vivo*, it was examined how the duration from the time when a mass was noticed to extraction (disease duration) was associated with the proportion of eosinophils to shadow cells and the presence or absence of bone formation and giant cells. The average disease duration was 13.9 years in cases with only shadow cells, while it was 2.7 years in cases with both eosinophils and shadow cells, and it was significantly longer in the former cases (*P* < 0.01). With regard to osteogenesis, the average disease duration was 11.5 years in cases with bone formation, while it was 0.8 years in cases without it, and it was significantly longer in the former cases (*P* < 0.001). The average disease duration was 4.0 years in cases with giant cells, while it was 7.1 years without them, and there was no significant difference between the former and latter cases. 

 Taken together, the present case with only shadow cells and bone formation was considered an old one. Bone formation is thought to appear subsequent to calcification [1], and Anneroth and Sigurdson mentioned that giant cells were osteoclast-type cells without phagocytic capacity in giant cell epulis and the stroma was rich in fibroblasts and osteoblasts, which differentiated into bone and osteoid tissue, eventually forming bone [25]. 

 Since dye affinity changed from eosinophilic to basophilic properties in the parenchyma just beneath the osteoclast-like multinuclear giant cells around the edge of the calcified tumor parenchyma, Miyahara et al. reported that the edge of the calcified layer was decalcified and subsequently osteoblasts were induced to form bone, and a mechanism similar to general bone remodeling was present even in ectopic osteogenesis [24]. 

 In the present case, disease duration and formation of giant cells and bone were discussed with references, but no giant cell was observed in any case at the early stage. Therefore, it was speculated that the component proportion of eosinophils to shadow cells changed as disease stages advanced, and as shadow cells became predominant, giant cells appeared in the stroma, which promoted osteogenesis subsequently to calcification. 

In conclusions, we reported a case of calcifying epithelioma that became an old lesion without symptoms for 40 years. In 33 cases reported in our country from the oral and maxillofacial region, including our case, pathological characteristics and disease duration were compared by clinical statistics. 

## Figures and Tables

**Table 1:** 

Case	Reporter	Reported year	Age	Sex	Region	Duration that the patients lived with symptoms (year)	Pathological findings	Bone formation	Giant cells	Tumor diameter (mm)
1	Suzuki	1978	12	F	Around the right auricle	1.67	Eosinophils + shadow cells	−	+	45 × 25 × 30
2	Goto	1983	6	M	Around the left auricle	0.5	Eosinophils + shadow cells	−	+	28 × 25 × 5
3	Daito	1985	27	M	Left parotid masseteric	0.17	Eosinophils + shadow cells	−	+	70 × 45 × 25
4	Aoki	1987	8	F	Right cheek	0.5	Eosinophils + shadow cells	−	+	11 × 17 × 13
5	Kurachi	1989	4	M	Right cheek	1	Shadow cell only	−	+	4 × 5 × 8
6	Hasegawa	1992	5	M	Left cheek	1	Eosinophils + shadow cells	−	+	20 × 15 × 9
7	Katsuyama	1995	7	F	Left mandibular angle	0.67	Shadow cell only	−	−	22 × 16
8	Ashiyama	1995	7	M	Right cheek	5	Eosinophils + shadow cells	−	+	20 × 12 × 12
9	Nishimura	1996	52	M	Left neck	0.08	Eosinophils + shadow cells	−	−	10 × 8
10	Nobumori	2002	5	F	Left cheek	0.08	Eosinophils + shadow cells	−	−	5 × 5
11	Suzuki	2004	56	M	Right mandibular angle	1	Eosinophils + shadow cells	−	+	20 × 10 × 10
12	Fujita	2005	24	M	Around the right auricle	0.08	Eosinophils + shadow cells	−	+	12 × 10
13	Fujita	2005	29	M	Left cheek	0.08	Eosinophils + shadow cells	−	+	20 × 20
14	Fujita	2005	13	M	Left neck	0.08	Eosinophils + shadow cells	−	+	15 × 10
15	Fujita	2005	21	M	Around the right auricle	0.17	Eosinophils + shadow cells	−	+	39 × 32
16	Miyahara	2007	68	F	Right cheek	0.5	Eosinophils + shadow cells	−	−	18 × 18
17	Miyahara	2007	20	F	Left neck	0.67	Eosinophils + shadow cells	−	+	21 × 22
18	Miyahara	2007	12	F	Around the right auricle	1	Eosinophils + shadow cells	−	−	15 × 13 × 6
19	Miyahara	2007	4	F	Left neck	1	Eosinophils + shadow cells	−	−	15 × 20
20	Miyahara	2007	21	F	Left mandibular angle	1.5	Eosinophils + shadow cells	−	−	25 × 15
21	Hasegawa	1983	30	M	Right neck	20	Eosinophils + shadow cells	+	−	15 × 13 × 6
22	Kawaguchi	1983	22	F	Around the right auricle	4	Shadow cell only	+	+	7 × 7 × 6
23	Tanaka	1985	41	F	Around the right auricle	20	Eosinophils + shadow cells	+	−	19 × 22 × 7
24	Kamakura	1991	23	F	Around the right auricle	13	Shadow cell only	+	+	40 × 28 × 15
25	Takano	1993	59	F	Left parotid masseteric	30	Shadow cell only	+	−	30 × 20
26	Takano	1993	38	M	Left parotid masseteric	30	Shadow cell only	+	−	20 × 13 × 7
27	Tsukamoto	1994	12	F	Left parotid masseteric	0.25	Eosinophils + shadow cells	+	+	50 × 28
28	Nishimura	1996	11	F	Around the left auricle	8	Shadow cell only	+	−	10 × 8 × 7
29	Fujita	2005	23	F	Around the left auricle	0.08	Eosinophils + shadow cells	+	−	7 × 8
30	Fujita	2005	27	F	Around the left auricle	5	Eosinophils + shadow cells	+	−	14 × 20
31	Maejima	2006	37	M	Around the right auricle	0.08	Shadow cell only	+	−	26 × 26 × 15
32	Miyahara	2007	20	F	Around the right auricle	4	Eosinophils + shadow cells	+	−	20 × 13
33	Our patient	2011	43	M	Around the right auricle	40	Shadow cell only	+	+	10 × 9
